# Moving Object Detection for Video Surveillance

**DOI:** 10.1155/2015/907469

**Published:** 2015-03-11

**Authors:** K. Kalirajan, M. Sudha

**Affiliations:** ^1^Department of Electronics and Communication Engineering, SVS College of Engineering, Coimbatore 642 109, India; ^2^Department of Electronics and Communication Engineering, Hindustan College of Engineering & Technology, Coimbatore 641 032, India

## Abstract

The emergence of video surveillance is the most promising solution for people living independently in their home. Recently several contributions for video surveillance have been proposed. However, a robust video surveillance algorithm is still a challenging task because of illumination changes, rapid variations in target appearance, similar nontarget objects in background, and occlusions. In this paper, a novel approach of object detection for video surveillance is presented. The proposed algorithm consists of various steps including video compression, object detection, and object localization. In video compression, the input video frames are compressed with the help of two-dimensional discrete cosine transform (2D DCT) to achieve less storage requirements. In object detection, key feature points are detected by computing the statistical correlation and the matching feature points are classified into foreground and background based on the Bayesian rule. Finally, the foreground feature points are localized in successive video frames by embedding the maximum likelihood feature points over the input video frames. Various frame based surveillance metrics are employed to evaluate the proposed approach. Experimental results and comparative study clearly depict the effectiveness of the proposed approach.

## 1. Introduction

Recently, several contributions have been proposed and successfully demonstrated for foreground detection and tracking. However, these algorithms need to resolve the difficulties such as radical changes and target drift encountered during tracking process. Main challenge involved in motion tracking algorithm is to estimate object motion as more precisely and efficiently as possible. Moving object detection is an important aspect in any surveillance applications such as video analysis, video communication, traffic control, medical imaging, and military service [1]. Usually video frames contain foreground as well as background information, in which the feature points in the region of interest are the foreground information and the remaining feature points are considered to be background information.

In general, video surveillance system involves two major building blocks such as motion detection and motion estimation. Object detection is the first and foremost step as it is directly influenced by the background information. Since there is considerable irrelevant and redundant information in the video across space and time, the video data need to be compressed at the earliest in video surveillance applications [2]. Compression can be achieved by minimizing the spatial and temporal redundancies present in the video. In earlier days, the video data is compressed either by reducing the size of the frame or by frame skipping with small degradation in video quality [3]. The 2D orthogonal transforms and motion compensation techniques are involved in recent video coding standards to remove the spatial and temporal redundancies. In the proposed method, 2D discrete cosine transform is used for video compression because of its highest energy compaction. The motion detection and motion estimation are the two major building blocks of video surveillance system [4]. In motion detection, the moving object is identified by extracting the changes in object boundaries whereas, in motion estimation, the motion vectors are computed to estimate the positions of moving objects [5]. The optimal motion vector is explored by finding the displacement of coordinates of the best match in a reference frame for the block in a current frame [6]. Optical flow vector is calculated using Horn-Schunck algorithm for moving object detection [7]. Since it assumes smoothness in the flow over the whole image frame, it is more sensitive to noise and unsuccessful under occlusion conditions [7]. The RLOF has excellent long-term feature tracking performance, but its computational complexity is more as compared to KLT [8]. The background subtraction [9] is one among the methods of extracting the foreground object for motion analysis in video surveillance. Nonstationary backgrounds and illumination changes are bottleneck problems in the background subtraction method [9]. In practice, the global constraints of optical flow based algorithms are violated which results in tracking error under cluttered environments. In most of the background subtraction methods, the object trackers are influenced by the background information which leads to false detection. Further, an effective classifier is required to discriminate the target in cluttered environments [10]. To overcome these limitations, a novel approach is presented in this paper which effectively detects the target in complex environments without background influences. The key contributions of this paper can be summarized as follows.

- In video compression, the input video frames are compressed by the 2D discrete cosine transform with acceptable blocking artifacts to reduce storage requirements.
- In object detection, the matching feature points are derived by calculating the correlation coefficients between compressed video frame and target template.
- Then the posterior probabilities are formulated and maximum likelihood densities are estimated by calculating the peak correlation coefficients over the entire image frame. These highly matching feature points are localized based on Bayesian rule.
- Finally, the matching feature points are localized in the successive video frames by embedding the maximum likelihood densities over the input frames.

## 2. Materials and Methods

**Figure 1:**
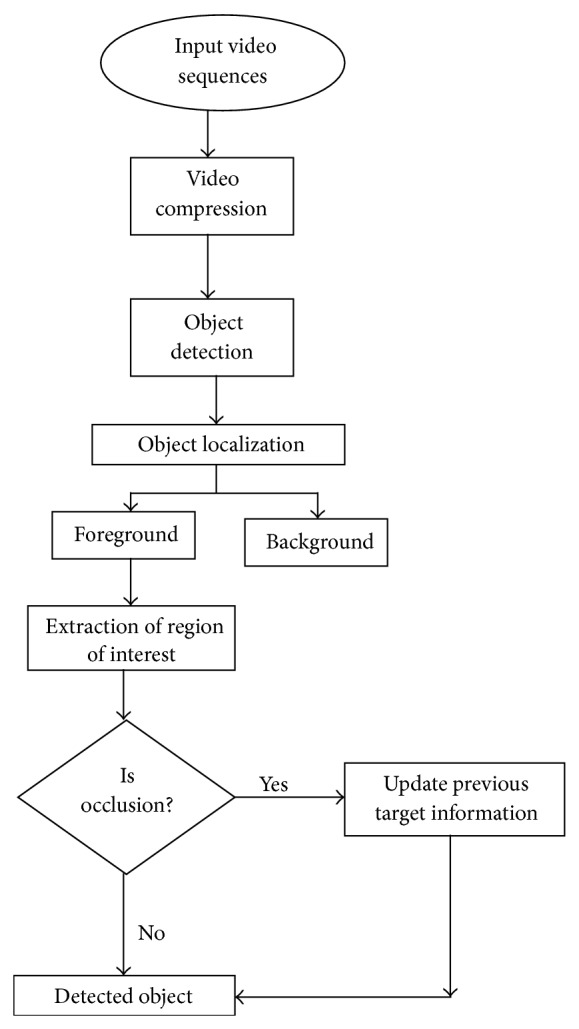
The flow diagram of proposed framework.

**Figure 2:**
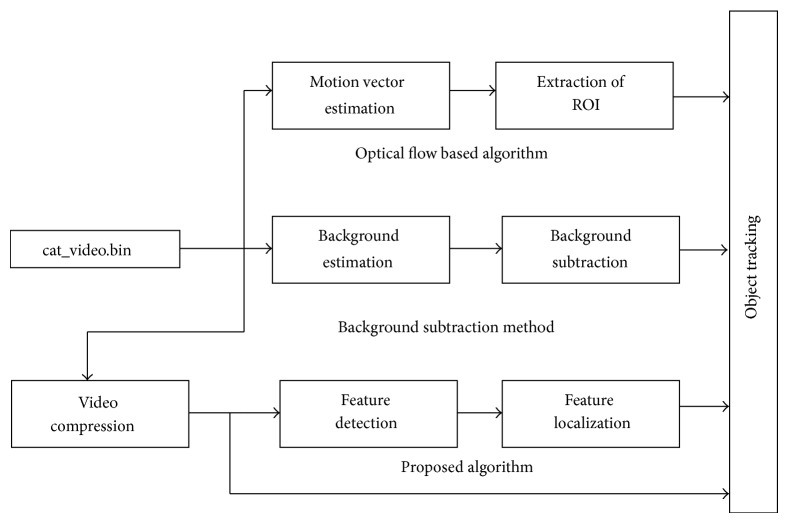
Simulink Model of proposed scheme.

The flow diagram and Simulink model of proposed framework are shown in Figures 1 and 2, respectively. The algorithm framework is divided into different parts including video compression, object detection and object localization.

### 2.1. Video Compression

In the first phase of proposed framework, the input video frames are compressed using block processing algorithm called 2D discrete cosine transform (DCT) [11]. Let *F*(*u*, *v*) be the transformed frame and let *f*(*x*, *y*) be the original frame. Consider an image frame with dimensions of (*M* × *N*), where *M* and *N* are the rows and columns involved in each image frame. The transformed and compressed image frames are estimated as follows:

(1) Fu,v=αuαv∑x=0M−1∑y=0N−1fx,ycos⁡2x+1uπ2M        ·cos⁡2y+1vπ2N,

(2) fx,y=∑u=0M−1∑v=0N−1αuαvFu,vcos⁡2x+1uπ2M    ·cos⁡2y+1vπ2N,

where

(3) αu=1M;u=01M;u=1,2,3,4,…,M−1;αv=1N;v=01N;v=1,2,3,4,…,N−1.

The two-dimensional discrete cosine transform computes the transform coefficients by dividing the entire frame into various subblocks of size (8 × 8) and applying the 2D DCT [11] over each individual subblock. The resulting coefficients are then simultaneously quantized and coded. Since most of these transform coefficients have small magnitudes, they can be entirely discarded with an acceptable error. The error between the original and compressed video frames is usually enumerated by the factors, namely, mean square error (MSE) and peak signal to noise ratio (PSNR) [2]. The MSE between two frames “*f*” and “*s*” is given by the following equation:

(4) $$\begin{array}{c} \text{MSE}=\frac{1}{N}\underset{i,j}{\sum }{\left(f\left(i,j\right)-s\left(i,j\right)\right)}^{2}, \end{array}$$

where *i* and *j* denote the sum of all pixels in the image frames and *N* is the number of pixels per frame. Compression ratio and PSNR are the best metrics to assess the performance of video compression techniques. Compression ratio tells us how much amount of storage space is reduced and it is the ratio of compressed frame size to the actual frame size, whereas PSNR gives information about how far the compressed image frame is similar to the original frame [12]. Higher PSNR results in better fidelity.

The PSNR can be calculated as follows:

(5) $$\begin{array}{c} \text{PSNR}=\frac{10\log\left(255^{2}\right)}{\text{MSE}}. \end{array}$$

By increasing the block size in 2D DCT, we can achieve better compression ratio. However, increase in block size degrades the quality of an image frame.

### 2.2. Object Detection

Object detection is mainly concentrated to detect the target position in each frame with coordinates, scale, and orientation. In object detection phase, the feature vectors are derived using 2D correlation. Correlation is one of the statistical approaches which provide a direct measure of the similarity between two video frames and it will not be influenced by illumination variation and object translations. However, it cannot cope with image rotation and scaling. The proposed model can further be extended to deal with an image rotation and scaling by incorporating the sophisticated object detection algorithm such as multiresolution analysis. In proposed Simulink model, the 2D correlation block computes the two-dimensional cross correlation between compressed frame and template frame. At each location, the cross correlation coefficient has inflated scores for matching feature points and deflated scores for others. Let *S*(*x*, *y*) be the compressed video frame with a dimension (*M* × *N*) and *T*(*x*, *y*) be the template frame with a dimension (*P* × *Q*). Cross correlation *C*(*i*, *j*) is calculated by using the following equation:

(6) $$\begin{array}{c} C\left(i,j\right)=\overset{\left(M-1\right)}{\underset{x=0}{\sum }}\overset{\left(N-1\right)}{\underset{y=0}{\sum }}S\left(x,y\right)\cdot T{\left(x+i,y+j\right)}^{*}, \end{array}$$

where 0 ≤ *i* < (*M* + *N* − 1); 0 ≤ *j* < (*P* + *Q* − 1).

### 2.3. Object Localization

In this phase, an effective classifier is constructed to classify the matched features points into foreground and background using Bayesian rule [4]. Let *S*(*k*, *t*) be the input image frame at time *t* in the position *k*, *T*(*k*, *t*) be the template frame, and *f*_*v*,*t*_ be the feature vector of target in the position *k* at time *t*. The posterior probability of feature vector that appears from the background at position *k* is calculated as follows:

(7) $$\begin{array}{c} P\left(\frac{f_{\text{back}}}{f_{v,t}}\right)=\frac{P\left(f_{v,t}/f_{\text{back}}\right)P\left(f_{\text{back}}\right)}{P\left(f_{v,t}\right)}, \end{array}$$

where *f*_back_ is the background and *P*(*f*_*v*,*t*_/*f*_back_) is the probability of feature vector *f*_*v*,*t*_ being observed as background. The prior probability of feature vector being identified at the position *k* is denoted by *P*(*f*_*v*,*t*_) and *P*(*f*_back_) is the prior probability of feature vector belonging to background. Similarly, the posterior probability of feature vector that appears from foreground at position *k* is calculated as follows:

(8) $$\begin{array}{c} P\left(\frac{f_{\text{fore}}}{f_{v,t}}\right)=\frac{P\left(f_{v,t}/f_{\text{fore}}\right)P\left(f_{\text{fore}}\right)}{P\left(f_{v,t}\right)}, \end{array}$$

where *f*_fore_ represents the foreground and *P*(*f*_*v*,*t*_/*f*_fore_) is the probability of feature vector *f*_*v*,*t*_ being observed as foreground. The prior probability of feature vector belonging to foreground is *P*(*f*_fore_). Thus, a probability map is constructed over the compressed video frames and the target is localized by searching the maximum likelihood density. When the template is centered at coordinates (*x*, *y*), the peak cross correlation coefficient indicates a good match of target location between compressed frame and the target template. Thus, the maximum likelihood density for foreground is estimated using the following equation:

(9) $$\begin{array}{c} \rho \left(f_{v,t},f_{\text{obj}}\right)=\max\left(\overset{\left(M-1\right)}{\underset{x=0}{\sum }}\overset{\left(N-1\right)}{\underset{y=0}{\sum }}S\left(x,y\right)\cdot T{\left(x+i,y+j\right)}^{*}\right). \end{array}$$

Thus, the posterior probability *P*(*f*_*v*,*t*_/*f*_fore_) can be estimated as follows:

(10) $$\begin{array}{c} P\left(\frac{f_{v,t}}{f_{\text{fore}}}\right)=C_{1}\rho \left(f_{v,t},f_{\text{obj}}\right), \end{array}$$

where *C*_1_ denotes normalization factor. Similarly, the matching feature points other than peak correlation coefficients in each image frame are considered as maximum likelihood densities for the background and are calculated as follows:

(11) ρfv,t,fback =1−∑x=0(M−1)∑y=0(N−1)Sx,y·Tx+i,y+j∗.

Hence, the posterior probability *P*(*f*_*v*,*t*_/*f*_back_) can be estimated as follows:

(12) $$\begin{array}{c} P\left(\frac{f_{v,t}}{f_{\text{back}}}\right)=C_{2}\max\rho \left(f_{v,t},f_{\text{back}}\right), \end{array}$$

where *C*_2_ represents normalization constant. At the end, the feature vectors {*f*_*v*,*t*_}_*n*=1,2,3,…,*N*_ can be classified as

(13) fv,t=ffore;for  Pfforegroundfv,t>Pfbackfv,tfback;otherwise.

## 3. Experimental Results and Discussions

This section elaborates the tracking results of proposed algorithm under challenging environments such as target variations, illumination changes, and occlusion conditions. The proposed algorithm is implemented in the testing platform of Pentium Dual-core CPU E5200@2.5 GHz and 2 GB RAM with MATLAB Simulink tool. The proposed scheme is tested on various video sequences including “cat_video.bin,” “FaceOcc2,” and “Dog1” with a frame rate of 30 fps, 28 fps, and 30 fps, respectively. This section is categorized into four parts such as performance analysis, quantitative evaluation, comparative study, and discussions.

### 3.1. Performance Analysis

In the proposed method, 2D discrete cosine transform, which is block based transform, is used for video compression because of its highest energy compaction. It simply decorrelates the similarities among the pixels. Initially, the given input frame is divided into several subblocks of size (8 × 8) and transform coefficients are obtained by applying 2D DCT over the entire subblocks of each frame. Then, transform coefficients with small magnitudes are discarded and the remaining coefficients are quantized and coded. Finally, the compressed image frame is obtained by applying inverse 2D DCT over the transformed frame. Since most of the DCT coefficients are removed for further processing, it greatly reduces the storage requirements. The compression ratio achieved by the proposed approach is enumerated in Table 1.  Table 2 illustrates the comparison of 2D DCT with other existing techniques. It can be seen that 2D DCT is superior to the other algorithms with acceptable blocking artifacts.

**Figure 3:**
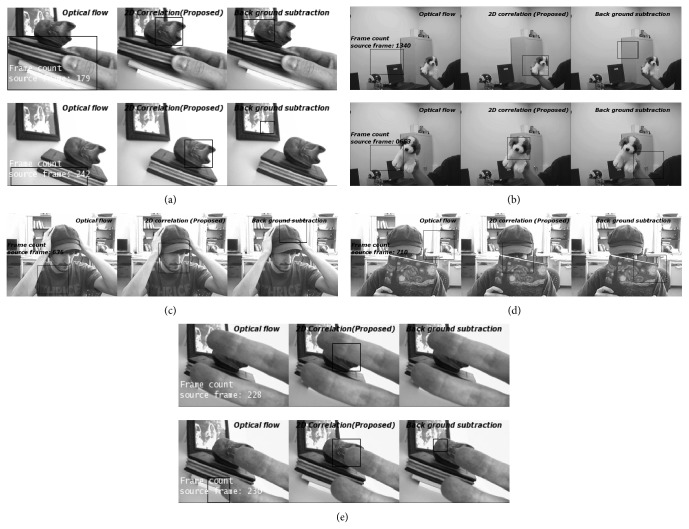
(a) Tracking results under target translations on cat_video.bin. (b) Tracking results under target variations on Dog1 data set. (c) Tracking results under target variations on FaceOcc2 data set. (d) Tracking results under complete occlusion on FaceOcc2 data set. (e) Tracking results under partial occlusions on cat_video.bin.

Figures 3(a)–3(e) show the tracking results of optical flow based Horn-Schunck algorithm, background subtraction, and proposed algorithm. For performance analysis of tracking process against the target translations and illumination changes, the frames 179 and 242 on “cat_video.bin” sequence are considered in Figure 3(a). Similarly, the frames 1340 and 663 on “Dog1” data set and 636 on “FaceOcc2” data set are considered in Figures 3(b) and 3(c), respectively. In all the frames, the existing approaches such as optical flow based Horn-Schunck algorithm [7] and background subtraction algorithm [9] are vulnerably deviated from the target and influenced by the background information. On the other hand, the proposed system captures the target more precisely without target drift.

#### 3.1.1. Occlusion Handling

Occlusion is one of the main challenges in object detection and tracking. Majority of the tracking systems are struggled to trace the target or even sometimes failed to follow the target during partial or complete occlusion conditions due to the unavailability of target information. Hence, it is mandatory to develop a robust algorithm to effectively cope with the partial and complete occlusions. Figures 3(d)–3(e) illustrate partial and complete occlusions. The test frames 228 and 230 on “cat_video.bin” video sequence and 710 on “FaceOcc2” data set are considered in Figures 3(d)–3(e) to validate the tracking performance under occlusion conditions. It is obvious that the proposed model is able to recognize the occluded target in all frames, whereas existing algorithms such as optical flow based algorithm and background subtraction method are not succeeded in occlusion conditions.

**Figure 4:**
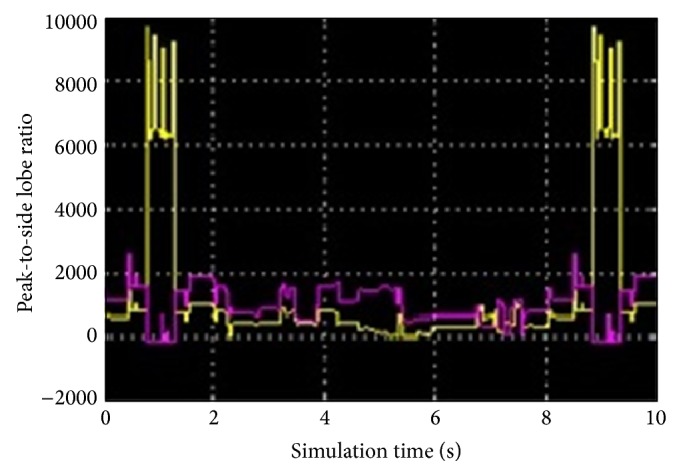
Estimated Peak-to-Side lobe Ratio (PSR).

Additionally, the proposed algorithm employs the peak-to-side lobe ratio (PSR) [13] to estimate the location of fully occluded target in “cat_video.bin” video sequence. In proposed scheme, the peak-to-side lobe ratio (PSR) is calculated as follows:

(14) $$\begin{array}{c} \text{PSR}=\frac{\left(C_{\max}-\overset{-}{x}\right)}{\sigma }, \end{array}$$

where *C*_max⁡_ is the peak correlation coefficient and $\overset{-}{x}$ & *σ* are the mean and standard deviation of the other coefficients.  Figure 4 shows the estimated PSR for the sequence “cat_video.bin” in which the yellow solid line specifies the calculated PSR values and the pink solid line shows the tracked position. It is observed that the strong peaks occur during the simulation time of 0.9 s to 1.2 s and 8.1 s to 9.33 s which point out that the target in frames 28 to 40 and 261 to 280 is completely occluded. In such cases, the proposed scheme incorporates the previous target features to maintain the target track and recaptures when it reappears. In contrast, the PSR facility was not found in optical flow based algorithm [7] and background subtraction method [9].

### 3.2. Quantitative Evaluation

**Figure 5:**
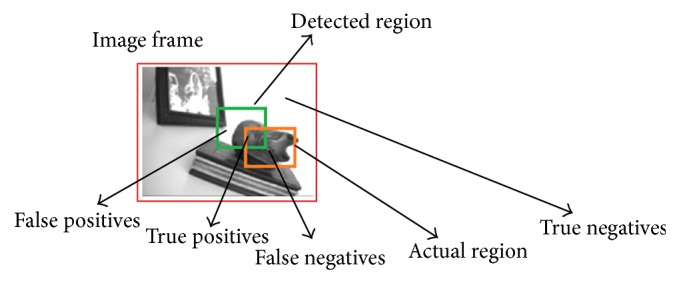
Illustration of frame based constraints.

Though the competency of proposed approach is proved by the above visual analysis, it is necessary to analyze the performance in quantitative manner.  Figure 5 illustrates the frame based constraints used for the evaluation of surveillance metrics. Here, the actual and detected regions of ground truth object (Gt) are represented by the brown and green colors bounding box, respectively. The fore grounds which are correctly detected are called true positives (Tp), whereas the undetected foregrounds are termed as false negatives (Fn). The falsely detected objects are referred to as false positives (F_s_).

The true negatives (Tn) are the objects which are not wrongly detected as background. In this paper, the detection is considered as success only when the bounding box overlaps the foreground object more than 50%. The performance metrics such as false alarm rate, precision, accuracy, and occlusion rate are computed using the following equations [14–17]:

(15) $$\begin{array}{c} \text{False}\;\text{alarm}\;\text{rate}=\frac{\text{Fp}}{\left(\text{Tp}+\text{Fp}\right)}, \end{array}$$

(16) $$\begin{array}{c} \text{Precision}=\frac{\text{Tp}}{\left(\text{Tp}+\text{Fp}\right)}, \end{array}$$

(17) $$\begin{array}{c} \text{Accuracy}=\frac{\left(\text{Tp}+\text{Fn}\right)}{\left(\text{Tp}+\text{Tn}+\text{Fp}+\text{Fn}\right)}, \end{array}$$

(18) Occlusion  rate =Number  of  successful  dynamic  occlusionsTotal  number  of  dynamic  occlusions.

The robustness of the object detection algorithm can be quantitatively evaluated by the above frame based metrics. For best performance, the metric false alarm rate must be lower whereas the metrics such as precision, accuracy, and occlusion rate should be higher. Relatively high scores in occlusion rate will indicate the success of object detection system in occlusion conditions.

### 3.3. Comparative Study

**Figure 6:**
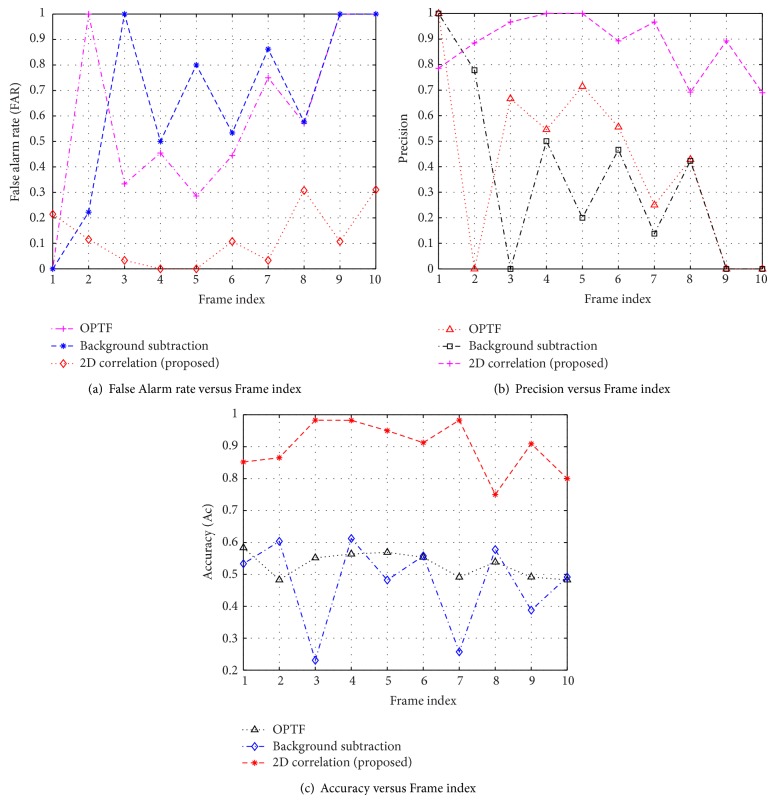
Comparative analysis of frame based surveillance metrics on “cat_video.bin” video sequence.

**Figure 7:**
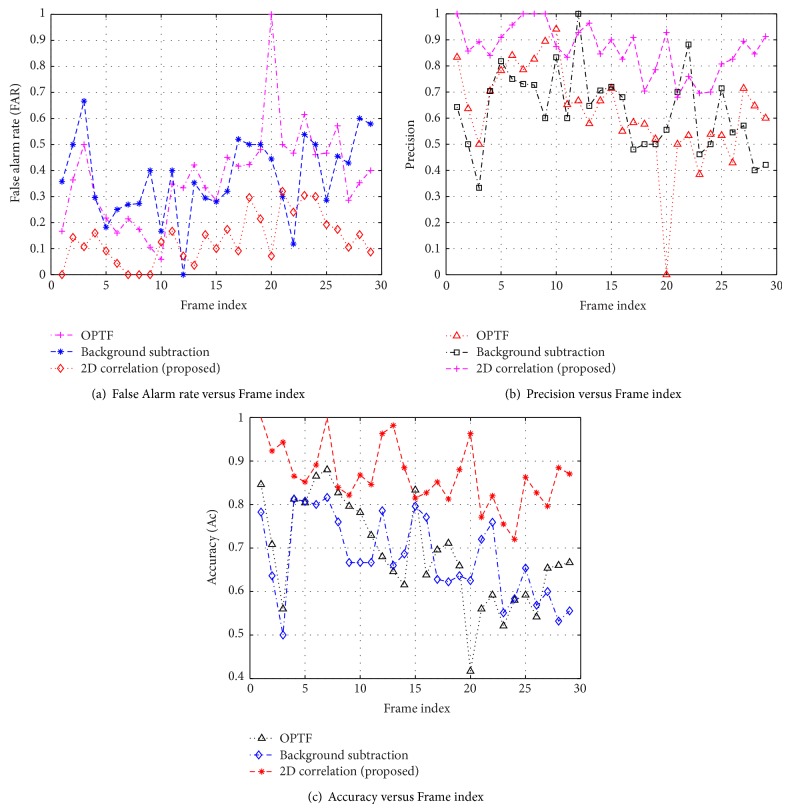
Comparative analysis of frame based surveillance metrics on “FaceOcc2” data set.

**Figure 8:**
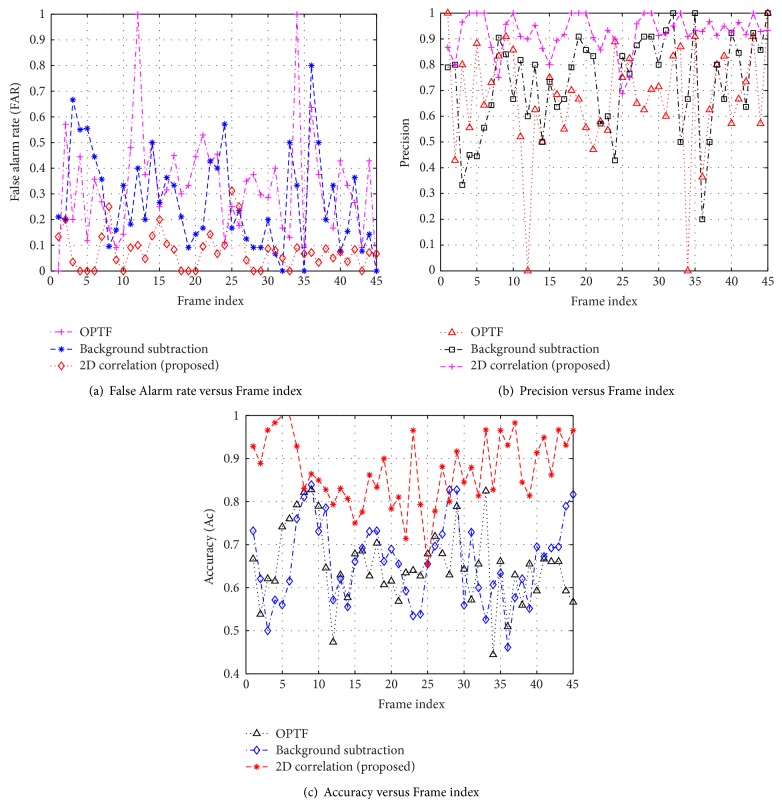
Comparative analysis of frame based surveillance metrics on “Dog1” data set.

For the comparative study, the existing algorithms [7, 9] are implemented using MATLAB Simulink tool and compared with the proposed approach. To demonstrate the robustness of proposed algorithm, the frame based surveillance metrics are deliberated and plotted in Figures 6, 7, and 8. It can be seen that the proposed scheme provides good results in all the surveillance metrics. The quantitative measures of surveillance metrics for optical flow [7], background subtraction [9], and proposed algorithm are summarized in Table 3. These metrics are obtained by averaging the individual metrics across the entire frame sequence. From the comparison, it is observed that the proposed scheme excelled under complex environments.

### 3.4. Discussions and Future Directions

Nonetheless, the proposed method is efficient in terms of all surveillance metrics, some issues yet to be addressed further. In view of rapid variations on both camera and target under dynamic environments, the target information is not enough for accurate object detection. Hence, the proposed algorithm does not perform well in dynamic backgrounds. In future, the research work will focus on deriving the most promising camera motion models and detection methods for online learning process. In proposed algorithm, 2D cross correlation is used for feature extraction to detect the presence of object in the given video frames. It is insensitive to illumination changes and object translations. However, it is sensitive to the image rotation and scaling which degrade the tracking performance.

In future, the performance of proposed method can further be improved by adding sophisticated feature extraction algorithm such as multiresolution analysis. Moreover, the target which is stationary for long time in video sequence misleads the object tracker into false detections. Future work will concentrate on this issue and try to improve the tracking performance.

## 4. Conclusion

In this paper, a robust algorithm has been proposed to detect and track the moving target in compressed video domain using statistical approach. In the proposed model, the input video frames are compressed using 2D DCT [11] to reduce the storage requirements with acceptable visual distortion. In proposed scheme, 2D DCT achieves better compression ratio (approximately 29 : 1) than other existing algorithms. In object detection, the matching feature points between the compressed frames and target template are estimated using statistical 2D correlation. In object localization, the posterior probabilities are formulated using Bayesian criterion [4] and the maximum likelihood densities are calculated by deriving the highest correlation coefficients. These maximum likelihood feature points are classified into foreground pixels and remaining matching feature points are classified into background based on the Bayesian rule. At the end, the classified foreground feature points are detected in successive image frames by rectangular bounding box. Experiment was conducted on the test sequences such as “cat_video.bin,” “FaceOcc2,” and “Dog1” and the performance was qualitatively analyzed. The proposed method effectively handles the challenging environments including target translations and partial or complete occlusions and detects the target when it reappears. Several surveillance metrics [14–17] are quantitatively evaluated and compared with the other algorithms such as optical flow based algorithm [7] and background subtraction method [9]. The comparative study based on the surveillance metrics evidently illustrates the tracking efficiency of proposed algorithm under complex environments. Future work will investigate the methods to improve the tracking performance in all other aspects.

## Figures and Tables

**Table 1:** Compression ratio of proposed method on various sequences.

Video sequence	Actual size (KB)	Compressed size (KB)	Compression ratio
cat_video.bin	4608	3258	29.29 : 1
FaceOcc2	15600	9719	37.69 : 1
Dog1	10824	8767	19 : 1

Average compression ratio			28.66 : 1

**Table 2:** Comparison of compression ratio of proposed algorithm with other algorithms.

Compression algorithms	Compression ratio
Intel PLV [18]	12 : 1
IBM photo motion [18]	3 : 1
Fractals [18]	10 : 1
Motion JPEG [18]	10 : 1
Proposed	28.66 : 1

**Table 3:** Comparison of surveillance metrics on various sequences.

Sequences	Methods	FAR	Accuracy	Precision	Occlusion rate
cat_video.bin	Optical flow [7]	0.5839	0.5308	0.4161	9.90
	Background subtraction [9]	0.6495	0.4734	0.3505	21.78
	Proposed algorithm	0.1229	0.8987	0.8771	53.47

FaceOcc2	Optical flow [7]	0.2256	0.7881	0.7744	26.06
	Background subtraction [9]	0.3361	0.7249	0.6639	31.75
	Proposed algorithm	0.0669	0.9003	0.9331	63.507

Dog1	Optical flow [7]	0.3283	0.6506	0.6717	—
	Background subtraction [9]	0.2730	0.6604	0.7270	—
	Proposed algorithm	0.0781	0.8705	0.9219	—
